# Undetectable Serum Level of Anti-Müllerian Hormone (AMH) in a Woman with an Unpredictable Hyper-Response During Controlled Ovarian Stimulation for an IVF-ICSI Program: Case Report

**DOI:** 10.3390/diseases13110379

**Published:** 2025-11-19

**Authors:** Omar Sefrioui, Modou Mamoune Mbaye, Ismail Kaarouch, Smahane Aboulmaouahib, Latifa Ahbbas, Omar Touzani, Noureddine Louanjli, Bouchra Ghazi

**Affiliations:** 1 African Fertility Clinic (AFC), Fertility Clinic, Medical Assistance for Reproduction and Fertility Preservation, Casablanca 20000, Morocco; ismail.fsr@gmail.com (I.K.); s.aboulmaouahib@gmail.com (S.A.); ahbbaslatifa@gmail.com (L.A.); laboratoireltr@gmail.com (O.T.); n.louanjli@gmail.com (N.L.); 2 Labomac, Laboratory for Medical Analysis and Biology of Human Reproduction, Casablanca 20100, Morocco; 3 Immunopathology-Immunotherapy-Immunomonitoring Laboratory, Faculty of Medicine, Mohammed VI University of Health and Sciences (UM6SS), Casablanca 82403, Morocco; bghazi@um6ss.ma; 4 Mohamed VI Center for Research & Innovation (CM6RI), Rabat 10112, Morocco

**Keywords:** polycystic ovary syndrome (PCOS), anti-Müllerian hormone (AMH), ovarian hyperstimulation syndrome (OHSS), in vitro fertilization (IVF)

## Abstract

Background/Objectives: A decrease in serum AMH is generally associated with low ovarian response in assisted reproductive procedures, whether or not in vitro fertilization (IVF) with intracytoplasmic sperm injection (ICSI) is performed. Methods: We report a case involving a 31-year-old woman who had never been pregnant and with irregular menstrual cycles. An ultrasound scan performed on the second day of the cycle showed several annular follicles, a high luteinizing hormone (LH)/follicle-stimulating hormone (FSH) ratio suggesting polycystic ovary syndrome (PCOS), and an undetectable serum level of AMH. Results: Despite these observations, she exhibited an unpredictable hyperresponse during controlled ovarian stimulation, followed by a failed pregnancy despite successful in vitro fertilization with ICSI and a good-quality thawed embryo transfer (4AA). Conclusions: This case highlights the challenges of relying solely on AMH as a predictive marker of ovarian response. Although AMH is widely used for assessing ovarian reserve and stimulation outcomes, its limitations become evident in atypical cases. The paradoxical hyperresponse observed here may result from alternative regulatory mechanisms influenced by elevated LH levels, enhanced gonadotropin receptor sensitivity, or local ovarian factors. This report underscores the need for a personalized, multidimensional approach combining hormonal profiles, ultrasound assessments, and clinical history to optimize stimulation protocols and mitigate risks such as ovarian hyperstimulation syndrome (OHSS). Such tailored protocols are essential for managing patients with complex profiles, particularly those with undetectable AMH levels. Further research is needed to explore the mechanisms behind these atypical ovarian responses, including the roles of genetic polymorphisms, inflammatory markers, and environmental factors. This case demonstrates the importance of cautious interpretation of AMH results and emphasizes the value of comprehensive evaluations in assisted reproductive technologies.

## 1. Introduction

The anti-Müllerian hormone (AMH) is a dimeric glycoprotein secreted mainly by the granulosa cells of the ovarian follicles. Its expression is influenced by the follicular development stage, with production predominantly in the pre-antral and early antral stages. AMH is widely used as a marker of ovarian reserve in women of childbearing age, surpassing other traditional markers such as basal FSH, inhibin B, and estradiol [1,2]. Its serum concentration generally decreases with age and is correlated with ovarian response during assisted reproduction (ART) stimulation [3,4].

Polycystic ovary syndrome (PCOS) is a common endocrine disorder characterized by menstrual irregularities, hyperandrogenism, and polycystic ovarian morphology [5]. This syndrome is often associated with infertility due to ovulatory dysfunction. AMH is generally high in patients with PCOS due to increased antral follicles [6]. The LH/FSH ratio is also used as a diagnostic biomarker, although its relevance may vary across populations [7]. The impact of PCOS on ovarian response to stimulation remains complex, with some patients having an excessive response, while others have unexpected ovarian resistance [8].

Although AMH is an established indicator of ovarian reserve and stimulation response, its limitations are increasingly being highlighted [9]. Atypical cases, where a discrepancy is observed between AMH levels and ovarian response, suggest that other factors, such as the sensitivity of gonadotropin receptors and local ovarian regulation, are involved in follicular physiology [10]. In addition, individual variations exist, particularly in patients with PCOS, who may have a strong ovarian response despite undetectable levels of AMH [11].

In this context, we report the unique case of a 31-year-old patient with clinical and hormonal characteristics suggestive of PCOS, including a high LH/FSH ratio, but an undetectable serum level of AMH. Despite these observations, she presented an exaggerated ovarian response during a controlled stimulation in vitro fertilization with intracytoplasmic sperm injection (ICSI). This case highlights the complexity of ovarian biology and the need to interpret AMH levels by considering other clinical and biochemical parameters for individualized patient management.

## 2. Case Report

We report on a couple who came to consult for primary infertility at the Fertility Clinic, Medical Assistance in Reproduction and Preservation of Fertility, African Fertility Clinic (AFC) in Casablanca, Morocco, on 30 April 2024.

Male partner: A 46-year-old smoker with moderate teratozoospermia based on WHO 2021 standards (Table 1).

The ultrasound performed on the second day of the cycle revealed two hyperechogenic structures measuring 14 mm in the right ovary and 11 mm in the left ovary. Hormonal analyses performed in the follicular phase showed a high LH/FSH ratio (greater than 3.5), with concentrations of 25.79 mIU/mL for LH and 7.25 mIU/mL for FSH. Levels of estradiol, triiodothyronine (FT3), and progesterone were 49.80 pg/mL, 5.20 pg/mL, and 2.86 ng/mL respectively. However, serum AMH was undetectable (<0.01 pmol/L) (Table 2).

The other tests performed showed normal thyroid function and no evidence of insulin resistance or hyperprolactinemia. The very low concentration of AMH, considering unexpected patient age and ultrasound observations, raised the possibility of endogenous interference (hemolysis, lipemia, or hyperbilirubinemia) in the sample analyzed. This was ruled out after a visual inspection of the sample. In order to confirm this value, repeated AMH assays were performed in several independent laboratories and the results remained constant, thus ruling out possible analytical variability.

Overall, the main diagnostic criteria for polycystic ovarian syndrome (PCOS) were present, including a high LH/FSH ratio, biochemical hyperandrogenism, anovulation associated with polycystic ovarian morphology, and slightly increased BMI, although it is not a risk factor regularly observed in patients with PCOS.

### 2.1. Ovarian Stimulation Protocol

After a full clinical evaluation, the couple was scheduled for IVF–ICSI fertilization. Controlled ovarian stimulation was initiated on the second day of the cycle following an antagonist regimen, with 300 IU/day (Gonal 150 IU + Pergoveris 150 IU, Merck, Rahway, NJ, USA) for 6 days. On day 8, ovulation was blocked with Orgalutran^®^ (0.25 mg/0.5 mL, Ganirelix, MSD, Organon Canada Inc., Kirkland, QC, Canada).

In response to the unpredictable ovarian hyperresponse, ovulatory initiation was performed with Decapeptyl (2 0.1 mg) to prevent the risk of hyperstimulation. An ultrasound performed on the day of the initiation revealed the presence of more than 25 follicles. Estradiol and progesterone levels were 2831 pmol/L and 2.86 ng/mL, respectively. The follicular puncture, performed under general anesthesia 37 h after induction, allowed recovery of 29 oocytes, of which 18 were mature (metaphase II), all injected by ICSI. Fresh embryo transfer was not performed. Eleven embryos, eight blastocysts on day 5 and three on day 6, were cryopreserved.

### 2.2. Frozen Embryo Transfer Cycle

Three months later, the patient returned to the center for a frozen–thawed embryo transfer cycle. Estrofem^®^ (Laprophan, Novo Nordisk A/S, Bagsværd, Denmark) was prescribed on the second day of the menstrual cycle: two tablets a day for 7 days and then three tablets a day for 5 days. After ultrasound examination showing an 11 mm-thick endometrium, treatment with Gestel^®^ and progesterone was initiated. Seven days later, an embryo transfer was performed using an Elliocath^®^ (Ellios, Laboratoires Ellios Bio Tek, Boulogne-sur-Mer, France) catheter.

Unfortunately, a pregnancy was not obtained 14 days after the transfer. Ten blastocysts remain vitrified for future trials.

## 3. Discussion

According to the literature, this case represents the first report of a woman with an AMH serum level lower than analytical sensitivity, but with an explosive, unpredictable, and inconsistent ovarian response with her baseline AMH. This calls into question the reliability of AMH in ovarian response assessment. Although low serum AMH levels are generally correlated with a low ovarian response, this relationship is not absolute, especially in women with undetectable levels. The ovarian response to exogenous stimuli may be influenced by other hormonal and environmental factors still poorly understood [12,13].

AMH is often considered the best marker to predict ovarian response during stimulation, surpassing FSH and other indicators such as age, estradiol, and inhibin B [14,15]. However, this case illustrates the limitations of AMH, especially in atypical situations. A paradoxical response, with an undetectable AMH but an explosive hyperresponse highlights the complexity of the mechanisms underlying ovarian stimulation. Similar cases were reported by the teams of Gubbels et al. and Grbavac et al., noting that AMH alone is not always sufficient to predict ovarian response [16,17].

One possible explanation is the role of local ovarian factors and the sensitivity of gonadotrophic receptors. In some women, especially those with PCOS characteristics, high levels of LH can increase granulosa cell activity and increase follicular reactivity despite low or undetectable AMH [18]. In addition, estradiol produced during stimulation can amplify follicular growth, underlining the need for an integrative approach to assess ovarian reserve [19]. Recent studies, including that of Melado et al., suggest pathophysiological clues to the mechanisms that may explain a preserved ovarian response despite low AMH [20]. Their study suggested that alterations in the expression of FSH and gonadotropins receptors could play a key role in this paradoxical response. Some patients with low AMH may have an increased sensitivity to exogenous gonadotropins, thus explaining their hyperresponse to stimulation. In addition, local factors such as increased production of intraovarian growth factors and altered signaling of the AMH pathway could influence follicular dynamics. These results support the idea that ovarian response is a multifactorial phenomenon that cannot be explained solely by serum AMH [20].

This case highlights the importance of personalized strategies in ovarian stimulation. Clinicians should not rely solely on AMH, but integrate a set of parameters including AFC, basal FSH, and LH/FSH ratios. Adjusting gonadotropin doses to the individual profile can prevent extreme responses, such as OHSS, even when AMH is low [21,22].

The use of Decapeptyl to trigger ovulation has minimized the risk of OHSS. However, a more comprehensive approach combining hormone and ultrasound monitoring could further improve management. Moreover, emerging research suggests that other biomarkers and genetic factors, such as polymorphisms of hormone receptors and inflammatory markers, may modulate ovarian response [23,24].

This case highlights the need to refine predictive models by integrating genetic and environmental data. The use of artificial intelligence could improve predictive capabilities by simultaneously analyzing multiple parameters [25]. The study of molecular pathways involved in follicular development could also reveal new targets for optimizing stimulation protocols [26].

The clinical implications are major: on the one hand, they reinforce the importance of a multidimensional approach in ART, combining biomarkers, hormonal analyses, and ultrasounds; on the other hand, they underline the need for individualized care, particularly for patients with an atypical profile such as undetectable AMH [27].

Finally, patient education is essential. Clinicians need to clarify the limits of current biomarkers and anticipate atypical ovarian responses in order to better tailor treatments. This case highlights the need for further research to improve understanding of underlying mechanisms and refine clinical strategies in ART [28].

## 4. Conclusions

This report highlights the importance of interpreting AMH results with caution, especially in patients with undetectable levels. Clinicians are advised to incorporate additional assessments, such as antral follicle count and individual hormone profiles, to customize stimulation protocols. This case also highlights the need for extensive research to explore underlying factors that may influence ovarian response in atypical patients. This research should include the impact of genetic variations, environmental parameters, and hormonal fluctuations in order to develop more reliable and robust predictive tools for reproductive biology professionals in their clinical practice. A multidimensional strategic approach could help improve outcomes and minimize complications associated with controlled ovarian stimulation.

The observations of this case also call for a reassessment of the current dependence on AMH as the single marker of ovarian reserve and response. The paradoxical response observed in this patient suggests the existence of alternative pathways influencing ovarian physiology that are not reflected by AMH alone. This highlights the need to integrate investigations at the molecular level, particularly on the role of the follicular microenvironment and receptor sensitivity, into clinical practice.

This case also highlights the importance of improving patient counseling and setting realistic expectations, especially for those with atypical profiles. Personalized medicine based on advanced diagnostics and predictive models based on artificial intelligence could revolutionize decision-making in reproductive care.

Finally, future studies should prioritize longitudinal monitoring of ovarian responses and outcomes in diverse populations, including those with PCOS-like characteristics with undetectable levels of AMH. This research would provide valuable insights to adapt interventions, reduce risks, and improve success rates of assisted reproductive technologies. By bridging the gap between clinical observations and biological mechanisms, a comprehensive approach to ovarian stimulation protocols could pave the way for safer and more effective fertility treatments.

## Figures and Tables

**Table 1:** Clinical and spermographic parameters of the male partner.

Category	Parameter	Observed Value	Reference Values (WHO 2021)
	Age	46 years	-
General	Smoking status	Smoker	Nonsmoking
	Concentration (million/mL)	12	>15
Spermogram	Progressive mobility (%)	25	>30
	Vitality (%)	50	>54
	Normal morphology (%)	3.5	>4
	Volume of semen (mL)	1.8	>1.4

**Table 2:** Clinical, biological, and outcome outcomes of managing a couple with primary infertility.

Category	Parameter	Observed Value	Reference Values
General information	Age	31 years	-
	BMI	27 kg/m^2^	18.5–24.9
	Menstrual cycles	Irregular	Regular
	Signs of hyperandrogenism	Absent	Absent
Echocardiographic parameters	Antral follicles (day 2)	14 mm (OD), 11 mm (OG)	-
Hormonal parameters	LH/FSH ratio	>3.5	<2
	LH	25.79 mIU/mL	2–10 mIU/mL
	FSH	7.25 mIU/mL	3–10 mIU/mL
	Total testosterone	45 ng/dL	20–80 ng/dL
	Estradiol (day 2)	49.80 pg/mL	<60 pg/mL
	Progesterone (day 2)	0.86 ng/mL	<1 ng/mL
	AMH	<0.01 pmol/L	1.5–4.0 ng/mL
	Thyroid function	Normal	-
	Insulin resistance	Absent	Absent
	Hyperprolactinemia	Absent	Absent
Results of stimulation	Oocytes retrieved	29 (18 metaphases II)	-
Result of the transfer	Cryopreserved embryos	11 (8 on day 5, 3 on day 6)	-
	Endometrium before transfer	11 mm	>7 mm
	Result of embryo transfer	Pregnancy not achieved	-

## Data Availability

Data supporting the reported results are available from the corresponding author.

## Abbreviations

The following abbreviations are used in this manuscript:

AFC	antral follicle count
AMH	anti-Müllerian hormone
ART	assisted reproductive technology
BMI	body mass index
FSH	follicle-stimulating hormone
ICSI	intracytoplasmic sperm injection
IVF	in vitro fertilization
LH	luteinizing hormone
OHSS	ovarian hyperstimulation syndrome
